# Combining QTL Analysis and Genomic Predictions for Four Durum Wheat Populations Under Drought Conditions

**DOI:** 10.3389/fgene.2020.00316

**Published:** 2020-05-06

**Authors:** Meryem Zaïm, Hafssa Kabbaj, Zakaria Kehel, Gregor Gorjanc, Abdelkarim Filali-Maltouf, Bouchra Belkadi, Miloudi M. Nachit, Filippo M. Bassi

**Affiliations:** ^1^Laboratory of Microbiology and Molecular Biology, Faculty of Sciences, Mohammed V University, Rabat, Morocco; ^2^ICARDA, Biodiversity and Integrated Gene Management, Rabat, Morocco; ^3^The Roslin Institute, The University of Edinburgh, Edinburgh, United Kingdom

**Keywords:** genomic selection, consensus map, drought, imputation, QTL analysis, fixed effect, consensus map, genotyping by sequencing (GBS)

## Abstract

Durum wheat is an important crop for the human diet and its consumption is gaining popularity. In order to ensure that durum wheat production maintains the pace with the increase in demand, it is necessary to raise productivity by approximately 1.5% per year. To deliver this level of annual genetic gain the incorporation of molecular strategies has been proposed as a key solution. Here, four RILs populations were used to conduct QTL discovery for grain yield (GY) and 1,000 kernel weight (TKW). A total of 576 individuals were sown at three locations in Morocco and one in Lebanon. These individuals were genotyped by sequencing with 3,202 high-confidence polymorphic markers, to derive a consensus genetic map of 2,705.7 cM, which was used to impute any missing data. Six QTLs were found to be associated with GY and independent from flowering time on chromosomes 2B, 4A, 5B, 7A and 7B, explaining a phenotypic variation (PV) ranging from 4.3 to 13.4%. The same populations were used to train genomic prediction models incorporating the relationship matrix, the genotype by environment interaction, and marker by environment interaction, to reveal significant advantages for models incorporating the marker effect. Using training populations (TP) in full sibs relationships with the validation population (VP) was shown to be the only effective strategy, with accuracies reaching 0.35–0.47 for GY. Reducing the number of markers to 10% of the whole set, and the TP size to 20% resulted in non-significant changes in accuracies. The QTLs identified were also incorporated in the models as fixed effects, showing significant accuracy gain for all four populations. Our results confirm that the prediction accuracy depends considerably on the relatedness between TP and VP, but not on the number of markers and size of TP used. Furthermore, feeding the model with information on markers associated with QTLs increased the overall accuracy.

## Introduction

Durum wheat (*Triticum durum* Desf., 2n = 4x = 28, AABB) is grown annually on over 17 million hectares worldwide, and it represents one of the bases of the Mediterranean diet. This region is the largest consumer of durum wheat products and the most significant durum import market (Soriano et al., 2017). The Mediterranean basin is subject to frequent droughts and their occurrence is expected to raise in the near future, with a significant negative effect on crop development and production (Xiao et al., 2018). Breeding for durum genotypes that have an improved yield and tolerance to drought remains one of the most strategic methods to protect the harvest of this crop (Habash et al., 2009; Tadesse et al., 2016; Kuzmanoviæ et al., 2018). The use of genomic models to analyze the main drought adaptation traits can be deployed to significantly accelerate the breeding effort. Genetic linkage map and QTL mapping are useful tools for discovering genomic regions associated with traits of interest (Zhang et al., 2018). However, the significance of the identified QTLs is often linked to the specific parents used and it rarely proved useful for deployment in large scale breeding. One method to control for this error is to perform QTL discovery in multiple populations at the same time. The first step to achieve this is the development of genetic consensus maps that allow to bridge the discovery across populations. In fact, the development of consensus maps has already been shown to not only bridge the information between populations, but also to increase marker density, improve genome coverage, provide a validation of the marker ordering, and reduce markers gaps due to the absence of polymorphism between two parents (Marone et al., 2012; Maccaferri et al., 2014). Multiple genetic linkage maps have already been developed for wheat, and consensus genetic maps have been constructed for hexaploid wheat (Somers et al., 2004; Wang et al., 2014) and durum wheat (Maccaferri et al., 2014, 2015). Furthermore, high-throughput DNA sequencing technologies have now enabled the deployment of reliable and affordable marker coverage via genotype-by-sequencing (GBS), a methodology that relies on restriction enzymes to reduce the amount of genome to be sequenced (Poland et al., 2012; Edae et al., 2017). Numerous recent studies have used this marker system to identify quantitative trait loci (QTL) associated with yield, agronomic traits, and physiologic traits in drought and heat-stressed environments (Acuña-Galindo et al., 2015; Sukumaran et al., 2016; Edae et al., 2017; Hussain et al., 2017; Mwadzingeni et al., 2017; Asif et al., 2018; Bhatta et al., 2018; Roselló et al., 2019), in order to pyramid these QTLs via marker-assisted breeding (Edae et al., 2014).

Genomic selection (GS) builds on the concept of QTL analysis, but it explores the whole genome seeking large and small allelic effects (Bassi et al., 2016). Because of its capacity to better handle complex traits with several small effect alleles such as grain yield (GY), GS is now becoming the methodology of choice for incorporation into breeding strategies (Dekkers and Hospital, 2002; Crosbie et al., 2003; Bassi et al., 2016). GS analyzes jointly all markers to explain the total phenotypic variance through the sum of the markers effects (Meuwissen et al., 2001). Once a model is trained, an effect is assigned to each marker-allele, and the ‘genomic estimated breeding value’ (GEBVs; Meuwissen et al., 2001) can then be calculated for each individual as the sum of its allelic marker effects. The set of individuals used to train the model has both phenotypic and genotypic available and it is defined as the ‘training population’ (TP). The set of individuals from which the selection is made is defined as the ‘breeding population’ (BP), and only genotypic data are collected for it. The ‘accuracy’ of the predicted GEBV is determined by the correlation between GEBV and the true breeding value (TBV) calculated phenotypically for a ‘validation population’ (VP), which is genotyped and phenotyped, but not used to train the model. The value for accuracy is used to determine the overall success of the GS approach. Therefore, it is important to maintain a high degree of accuracy, and hence to use a TP that best fits the BP. The degree of relatedness between the two populations is often a good predictor of the accuracy that will be achieved. Cross-validation is used to train and develop the prediction models using different sampling techniques in the TP data sets ahead of estimating the GEBVs in the VP. The idea behind this approach is that breeders can derive predictions of the breeding value of an experimental line even before the line has been tested in the field. In turn, this would allow to make decisions on the use of the lines for yield testing or crossing already during the earlier generations (Crossa et al., 2010; Heffner et al., 2011; Bassi et al., 2016).

However, the integration of QTL analysis and GS remains severely understudied. In the present study, four recombinant inbreed lines (RILs) of durum wheat with different level of relatedness were field tested across environments. QTL analysis was performed for GY and TKW and the same populations were then used to assess different GS models for the two traits. The two methods were then combined by fixing the effect of the marker underlying the QTLs into GS models, to reveal a steep increase in the overall accuracy.

## Materials and Methods

### Mapping Populations

Four F_9_-derived RILs mapping populations were obtained by random selection of 200 individual durum spikes from each population at the F_4_ generation, followed by single seed descent to F_9_. At this generation, the individual plants were sampled for DNA extraction, and the seeds of each individual plant bulked. A different number of individuals for each population was then multiplied and used for yield trial to resemble the typical unbalanced dataset used by breeders. The four durum wheat crosses combining ICARDA’s elite lines were: Icamor/Gidara2 (IC; 115 RILs) developed by combining the *Hessian fly* resistance of Icamor (F413J.S/3/Arthur71/Lahn//Blk2/Lahn/4/Quarmal) with the high yield potential of Gidara2 (Stojocri/Omrabi3) (see Bassi et al., 2019 for more details); the second population was Jennah Khetifa/Cham1//T.dicoccoides600545/2^∗^Omrabi5 (DRO; 197 RILs) designed for pyramiding the drought tolerance of the Tunisian landrace Jennah Khetifa, wild emmer, and the ICARDA most successful variety Omrabi; the third population was SW Algia//Gidara1/Cham1 (SW; 93 RILs) aimed at incorporating the *Septoria tritici* resistance of the Tunisian landrace SW Algia with Gidara1; the fourth population was Omrabi3/Omsnima1//Gidara2 (YG; 145 RILs) aimed at combining drought tolerance and yield potential. As indicated, these populations all have sibling relationships with Omrabi, Cham 1, and Gidara used as parental lines. Additional details are reported in Table 1.

**TABLE 1:** Cluster analysis of the genetic diversity among four mapping populations using discriminant analysis of principal components (DAPC) with *k* = 4, their pedigrees, and maps features.

Pedigree	Individuals	Markers	Total length (cM)	Marker density (cM/Marker)
IC: Icamor/Gidara2	115	646	1720.1	5.3
DRO: Jennah Khetifa/Cham1// T.dicoccoides600545/2*Omrabi5	197	2291	1922.5	1.2
SW: SW Algia//Gidara1/Cham1	93	1212	1795.3	1.8
YG: Omrabi3/Omsnima1//Gidara2	145	521	1683.8	6.1

### Field Trials

Field trials were conducted during the 2014–2015 growing season. The experimental design used at all stations was an augmented complete block design with four common repeated checks, and a block size of 24 entries. The trials were conducted at three drought prone stations in Morocco (Supplementary Figure S1): Jemaat Shaim (JSH; 32°21′0′′ N and 8°51′0′′ W), Marchouch (MCH; 33°34′3.1′′ N and 6°38′0.1′′ W) and Sidi el Aidi (SAD; 33°9′36′′ N and 7°24′0′′ W); and one irrigated station in Lebanon: Terbol (TER; 33°48′29′′ N and 35°59′22′′ W) (Table 2 and Supplementary Figure S1). All RILs and their parents were planted in plots of 4.2 m^2^ at a seeding rate of 280 plants per m^2^. The YG population was planted in MCH, JSH, SAD and TER; the DRO population was also planted in all stations except TER; the IC population was sown in two stations MCH and TER; the SW population in just MCH. Agronomic practices were done following standard procedures, with 80 units of nitrogen provided in 2 equal splits, and 40 units of potassium and phosphorous before planting. Weeds were control by tank mixtures of Derby and Pallas. Days to heading (DTH), days to maturity (DTM), plant height (PLH), and spike density per m^2^ (SPK) were recorded in MCH and TER. At maturity, 3 m^2^ of the plot were combine harvested and the weight was converted to grain yield as Kg ha^–1^. At all stations except SAD, 1,000 kernels were weighted on a precision balance to derive 1,000-kernels weight (TKW) and express it in grams (g).

**TABLE 2:** Description of the field testing environments during the 2014–2015 season.

Code	Site	Country	Coordinates	Altitude (m)	Soil type	Climate	Moisture	Annual rainfall (mm)
MCH15	Marchouch	Morocco	33° 34′ 3.1′′ N, 6° 38′ 0.1′′ W	398	Clay vertisol	Mediterranean/warm temperate	Rainfed	449
SAD15	Sidi el Aydi	Morocco	33° 9′ 36′′ N, 7° 24′ 0′′ W	226	Vertisol	Mediterranean/hot and temperate	Rainfed	237
JSH15	Jemhâa Shaim	Morocco	32° 21′ 0′′ N, 8° 51′ 0′′ W	196	Calcic Cambisols	Hot steppe	Rainfed	270
TER15	Terbol	Lebanon	33° 48′ 29′′ N, 35° 59′ 22′′ W	897	Chromic Vertisols	Mediterranean/temperate	Sprinkle	559

### DNA Extraction and Genotyping

Leaf samples obtained from F_9_ plants were freeze-dried and used for C-TAB DNA extraction. DNA quality was assessed on agarose gel and it was then equilibrated to 100 ng. The DNA was shipped to the Poland lab at Kansas State University for genotyping by sequencing following the protocol of Poland et al. (2012). Briefly, two restriction enzymes (*Pst*I and *Msp*I) were used for genome complexity reduction, followed by 96-multiplex sequencing by bar coding. Low-quality data filtering was carried out according to the following rules: heterozygous calls not superior to 2%, maximum of 30% missing data, and a minor allele frequency superior to 10%.

### Consensus Map Procedure

Individual linkage maps for each population were constructed using the statistical software Carthagene v. 1.2.3 (De Givry et al., 2005) and QTL IciMapping V4.1 (Meng et al., 2015). First, all marker sequences were aligned to the available bread wheat genome assembly (Winfield et al., 2016; The International Wheat Genome Sequencing Consortium [IWGSC], 2018) by BLAST with an identity cut-off of 98% (1 SNP variant) and *E*-value of 5e^–25^. The *squeeze* function of Carthagene was used to eliminate markers that were wrongly ordered at LOD of 5 based on the genome alignment, followed by *flip* with window size of seven, LOD of 3, and zero iterations to determine the most plausible order of markers within each window. This framework map contained correctly aligned markers along the map and several unassigned markers. In QTL IciMapping, the framework markers were *anchored* while the unassigned markers were not. The *by anchor order* algorithm was used to assign to the different linkage groups the unassigned markers at a set LOD of 5, and then order them based on the position of the framework markers. This operation was then repeated using the newly developed framework map and reducing the LOD to 3. This methodology defined four individual genetic maps for each population.

The construction of the consensus map was performed chromosome by chromosome using the c*onsensus map from multiple linkage maps sharing common markers* (CMP) function of QTL IciMapping. First, by re-grouping markers at a distance of less than 20 cM to obtain one group for each chromosome, followed by the *by anchor order* option to measure the genetic distances between markers along the consensus map based on their relative positions on each individual map. Markers were then ordered based on their consensus map position in an Excel file. In several cases, a marker polymorphic in one population might be monomorphic in another. To avoid linkage distortions, the monomorphic scores were set to missing. At this point, imputation was done using AlphaIMpute option HMM (Hickey et al., 2012; Antolin et al., 2017) and confirmed with the BIP function of QTL IciMapping (Zhang et al., 2010).

### Data Analysis and QTL Mapping

Statistical analysis of the phenotypic data was performed using the R software version 3.4.3 and Genstat program version 18. Best linear unbiased estimates (BLUEs) were estimated across all environments, assuming fixed effects for the genotype from a linear mixed-effects model using R package *lme4* (Bates et al., 2015; R Core Team, 2017).

The discriminant analysis of principal components (DAPC), was performed using the ‘adegenet’ package 1.4-1 (Jombart et al., 2010) in R studio V 3.4.3 (R Core Team, 2017). With DAPC, the hierarchical clustering among populations was determined by applying the R based package “hclust.”

QTLs were searched for each individual population in each individual environment via composite interval mapping (CIM) analysis using R/qtl (Broman et al., 2003). The *cim* function was set to five markers covariates and a window size of 10 cM. LOD thresholds were calculated from QTL IciMapping by BIP functionality using 1,000 permutations with a maximum type 1 error probability of 0.05. Only QTLs that appeared at least in two environments and two populations were considered as valid. The distribution of QTLs and the marker density of the consensus and individual population maps were graphically presented on the fourteen chromosomes of durum wheat by a “Circos plot” using R/shiny application (Yu et al., 2018).

### Genomic Prediction Modeling

A total of four genomic models were tested as a first step in this study:

(i) a baseline additive model without interactions of genotypic effect (G), environmental (E) effect, and error (ε) (G+E + ε).
(ii) a baseline multi-environment model (G+E + GxE + ε), which assumed interactions between the G and the E.
In both these models, all the effects were assumed to be random with a normal distribution N(0, σ) where σ is the term variance
(iii) the third model was a marker (M) effect model (G+E + GxE + M + ε), where the genotype effect is substituted by an approximation of the genotype’s genomic value expressed as a regression on marker covariates.
In this case the model assumes that the genotype’s genomic value follows a normal distribution N(0, G σ_*g*_) where σ_*g*_ is the genetic variance and G is genomic relationship matrix.
(iv) the last model is the marker × environment model (G+E + GxE + M + MxE + ε) where the marker effect is composed by an effect common to all environment (main effect) plus a random deviation specific to a particular environment (Lopez-Cruz et al., 2015).

Testing of the different models’ accuracies was done using DRO, IG and YG populations independently, and setting as cross-validation 80% of the individuals as TP and 20% as VP. The accuracies within and across environments were then calculated as a measure of good fit. The BGLR package (Pérez and de Los Campos, 2014) was used to run all models above from (i) to (vii) by Bayesian ridge regression (BRR) using 10,000 iterations and 5,000 burn in, and 50 replications (de los Campos et al., 2009, 2013). This model induces homogeneous shrinkage of all marker effects toward zero and yields a Gaussian distribution of marker effects. The 50 replications were used to define statistical differences between model accuracies following a one factor ANOVA.

The GxE + MxE model (iv) was selected and used to test additional hypothesis:

(v) the effect of markers number was investigated by comparing predictions using 100, 80, 60, 40, 20, and 10% of the total marker set in combination with reducing the TP population size to 20, 50, and 75% for GY and TKW. The TP individuals were selected randomly in 50 replications, and one factor ANOVA was used to determine significant differences.
(vi) the prediction accuracy of using half sibs vs. full sibs as TP was compared. Each population was set as TP for all others and itself using the whole population as TP and the whole other population as VP.
(vii) to compare the value of MAS and GS, the prediction accuracy was calculated using 50% as TP and 50% as VP for all markers, only markers associated with major effect QTLs, with 44 and 27 markers for GY and TKW, respectively, and by removing these markers linked to QTLs from the set. The TP individuals were selected randomly in 50 replications, and one factor ANOVA was used to determine significant differences.
(viii) the rr-BLUP package v4.6 (Endelman, 2011) was used to run a mixed model estimating the accuracy gain when using markers underlying the QTLs as fixed effects, and the remaining markers as random effects. For this analysis ten random subsets of 50% TP and 50% VP were selected in each population separately (DRO, IG, SW, and YG). QTL analysis was conducted again for each TP subset following the method described above. Those markers that resulted as underlying QTLs in each TP subset were fixed in the model. One factor ANOVA was run for the ten replicates of each population to determine significant differences.

## Results

### Phenotypic Evaluation

**FIGURE 1:**
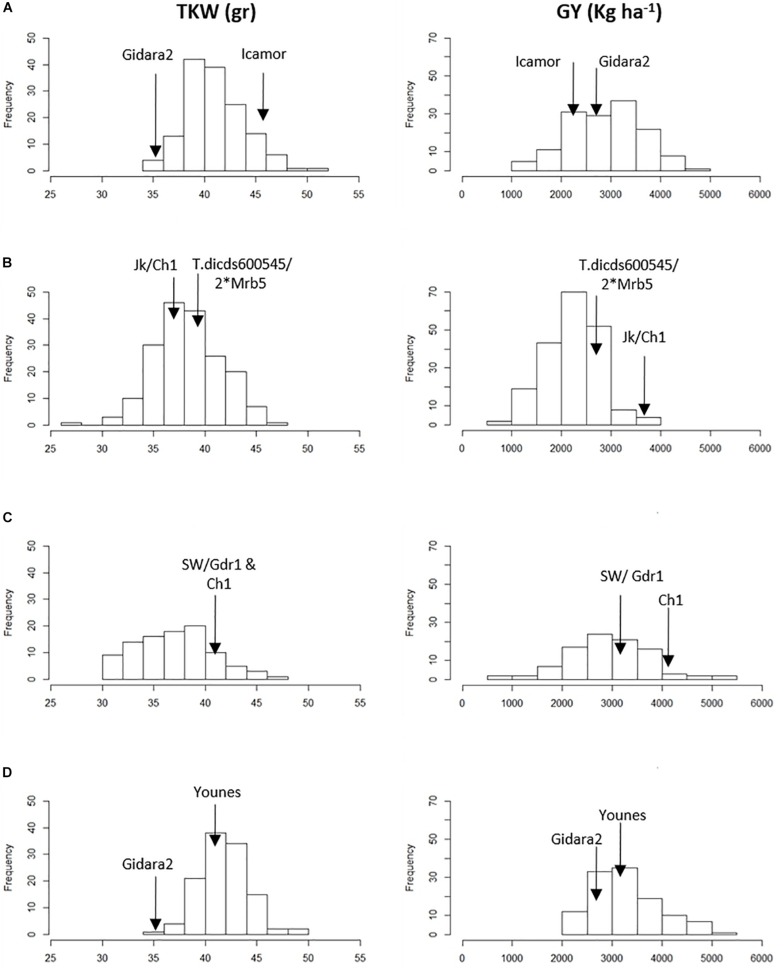
Frequency distribution of 1,000 kernel weight (TKW) and grain yield (GY) in the parents and the four RIL populations. **(A)** IC, **(B)** DRO, **(C)** SW, and **(D)** YG.

Analysis of variance (ANOVA) showed significant differences for genetic (G) effect (*p* < 0.05) for all the traits across environments, indicating good levels of phenotypic within each population (Table 3 and Figure 1). The genotype by environment interaction (GxE) effect was also significant (*p* < 0.05). The combined BLUE of TKW and GY differed greatly between the two parental lines of the four populations, displaying a normal distribution within RILs populations (Figure 1). Gidara 2 and Jk/Ch1 parents in populations IC, DRO and YG had smaller values of TKW than the average, whereas the Icamor parent in population IC had the maximum value (44 g). Similarly, for GY, Gidara 2 had a smaller value than the average GY, same for the parents Icamor and Younes. Cham1 parent of population DRO and SW had the highest recorded GY of this experiment. The population YG had the highest average TKW and GY. Among the four RILs populations, 50.2 g was the highest value recorded for TKW found in IC, and 3,304 kg ha^–1^ the highest GY for YG.

**TABLE 3:** Rate of genetic effect across environments of four populations (IC, DRO, SW, and YG) for DTH, DTM, PLH, SPK, TKW, and GY and genotype by environment interactions (GxE) effects.

Pop	GY across env.		DTH			DTM			PLH			SPK	TKW		
	GxE	G	MCH	SAD	TER	MCH	SAD	TER	MCH	SAD	TER	MCH	MCH	TER	JSH
IC	–	0.93*	0.44*	–	0.74*	0.94*	–	0.85	0.93*	–	0.89*	0.95	0.85*	0.99*	–
DRO	0.45*	0.53*	0.90*	1.00*	–	1.00*	0.86*	–	0.99*	0.98*	–	0.97*	0.95*	–	0.97*
SW	–	0.81*	0.94*	–	–	0.74	–	–	0.96*	–	–	0.89	0.97*	–	–
YG	0.63*	0.36*	0.90*	–	0.93*	0.92*	–	0.76*	1.00*	–	0.89*	0.99*	0.95*	0.93*	0.98*

*Significant at 0.05 probability level; −, not available data, GxE, genotype by environment interaction effect; G genetic effect; DTH, days to heading; DTM, days to maturity; PLH, plant height; SPK, spike density; TKW, 1000 kernel weight; MCH, Marchouch; SAD, Sidi el Aidi; TER, Terbol; JSH, JemaatShaim.

### Individual and Consensus Linkage Maps

The GBS process resulted in 22,117 marker calls. Among these, 4,909 matched the curation criteria and were tentatively ordered via genetic mapping. The individual genetic maps contained 646 polymorphic markers covering 1,720.1 cM for the IC population, 2,291 markers spanned 1,922.5 cM for DRO, 1,212 markers were mapped along 1,795.2 cM in SW, and 521 markers over 1,683.7 cM for YG (Table 4 and Supplementary Table S1). The final consensus map incorporated 3,202 markers assigned to 14 linkage groups corresponding to 1,883 unique loci, and spanned a total genetic distance of 2,705.7 cM, with a density of one marker each 0.85 cM (Table 4). The A genome, harbored 1,104 markers, covering a linkage distance of 1,133.8 cM, and the B genome 2,098 markers spanning a linkage distance of 1,572 cM. The largest chromosome was 2B, consisting of 540 markers and covering a genetic length of 243.5 cM, while the smallest chromosome in the map was 4A, covering a genetic length of 101.7 cM and consisting of 209 markers. The average size of markers gaps in the consensus map was 22.1 cM. The consensus map across four populations includes 550 RILS lines. Genetic diversity analysis revealed close kinship between IC and DRO, a lower relatedness with SW, and limited kinship to YG (Table 1).

**TABLE 4:** Characteristics of the consensus map.

Chr.	Markers	Loci	Length (cM)	Marker density (cM/Marker)	Size of largest gap (cM)
1A	118	72	138.8	1.2	26.7
1B	257	106	228.5	0.9	24.9
2A	220	164	135.6	0.6	17.9
2B	540	361	243.5	0.5	16.6
3A	146	74	199.5	1.4	21.1
3B	302	189	238.1	0.8	32.6
4A	209	130	101.7	0.5	6.3
4B	197	125	208.3	1.1	29.9
5A	105	38	217.5	2.1	29.7
5B	302	162	245.0	0.8	16.6
6A	162	75	171.8	1.1	17.5
6B	246	155	181.9	0.7	16.6
7A	144	80	168.9	1.2	20.8
7B	254	152	226.6	0.9	31.7
A genome	1104	633	1133.8	1.0	29.7
B genome	2098	1250	1572.0	0.7	32.6

### QTL Analysis

**FIGURE 2:**
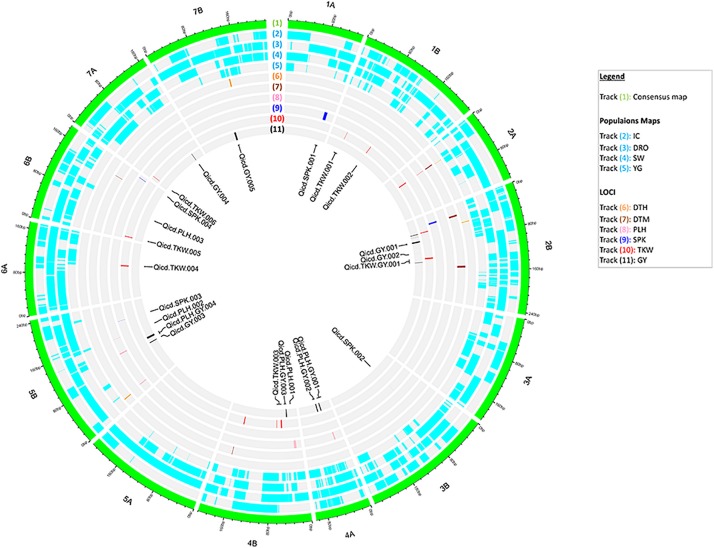
Circos representation of the consensus map with aligned the marker density within each population, and the identified QTL for all traits. From outer to inner layer: **(1)** the consensus map with 14 chromosomes of durum wheat; **(2–5)** the genetic maps of the four populations: IC, DRO, SW and YG; **(6–11)** distribution of significant markers identified via QTL analysis for DTH, DTM, PLH, SPK, TKW, and GY. In the center, labels for QTLs of PLH, SPK, TKW, and GY independent from flowering time.

The identified genetic and phenotypic variations were combined via QTL analysis across the 550 RILs for all measured traits. Significant QTLs were detected for all traits as summarized in Figure 2 (Supplementary Tables S2, S3). A total of 31 QTLs were detected across the four populations, explaining from 3.9 to 81.3% of the PV and LOD diverging from 3.7 to 43.5. Six QTLs were found to be associated with GY and independent from the flowering time. In particular, on chromosomes 2B, 4A, and 5B the four independent populations identified consistently the same GY QTL. Six QTLs were detected for TKW on chromosomes 1B, 4B, 6A, 6B, and 7A, explaining 4.7–15.9 of PV and with maximum LOD of 6.1. Interestingly, loci controlling TKW were found to be also associated to GY on chromosome 2B, explaining 8.6 and 4.8% of PV, and LOD of 4.7 and 4.3 respectively.

### Genomic Prediction: Identification of the Best Fitting Model (*i, ii, iii, iv*)

**FIGURE 3:**
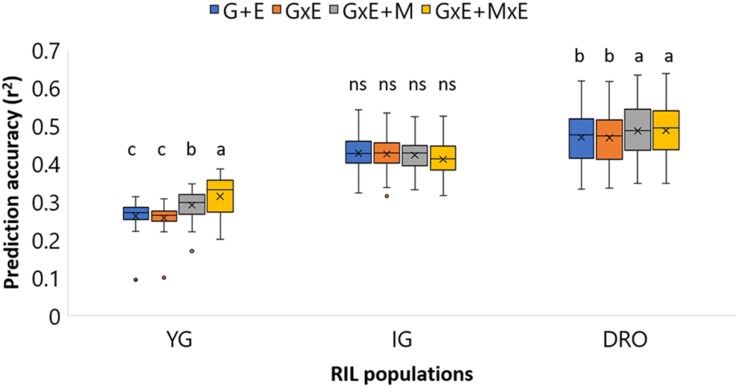
Prediction accuracy for grain yield (GY) in YG, IG, and DRO populations using four different statistical models. G+E, genotype + environment effect; GxE, genotype by environment interaction; GxE + M, genotype by environment interaction + markers effect; GxE + MxE, genotype by environment interaction + markers by environment interaction. The horizontal line represents the average, the square indicates the 2nd and 3rd quartiles, the whiskers represent the 1st and 4th quartiles, the cross the median, and the dots are outliers. The letters indicated classes determined via LSD.

Four statistical models (*i, ii, iii, iv*) were tested to determine the best model to be used for each population (Figure 3). Non-significant differences could be identified for the IG population with average accuracies that ranged from 0.42 to 0.41. For DRO, the incorporation of the M effect resulted in a significant increase in accuracy from 0.47 to 0.49. The YG population was the most sensitive to the change of model ranging from 0.27 for models without M (*i* and *ii*), to 0.30 for model *iii*, to 0.33 for model *iv* incorporating GxE + MxE. Following these results, the model incorporating GxE + MxE was chosen to be the best suited for all three populations. For the SW population phenotypic data were available only for one environment, therefore a model using only markers effect (*iii*) was used to run genomic predictions for this population.

### Genomic Prediction: Effect of Reducing TP and Marker Size (*v*)

**FIGURE 4:**
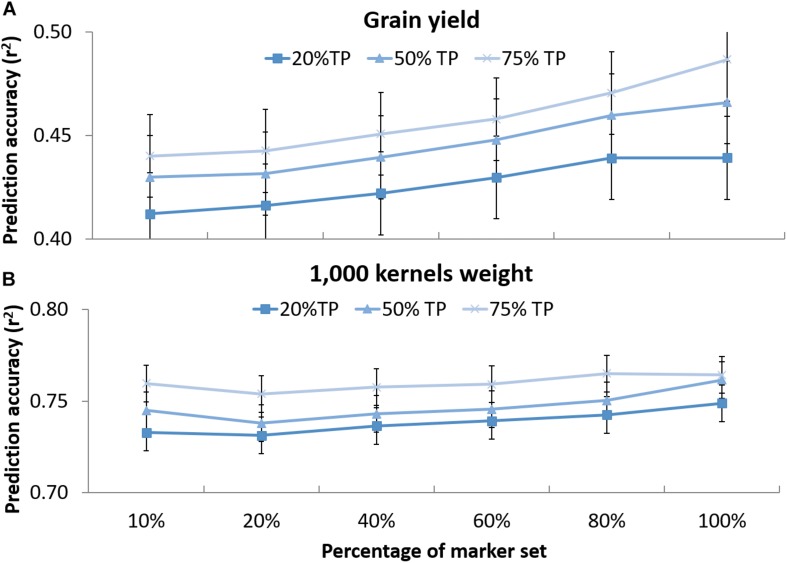
Prediction accuracy for grain yield **(A)** and 1,000 kernel weight **(B)** using different randomly selected sub-sets of markers in decreasing order: 320 (10%), 640 (20%), 1,281 (40%), 921 (60%), 2,562 (80%), and 3,202 (100%) tested on DRO population using 20, 50, and 75% of the whole population as training set (TP) to predict the rest of the population (VP). The whiskers represent the standard errors.

The effect of marker number and TP size on prediction accuracies was tested for GY and TKW (v). Figure 4 shows that when decreasing the number of markers from 3,202 to 320, a slight decrease in prediction accuracies was observed for the different set of TP. For GY, the reduction of markers caused a shift from 0.44 to 0.41 accuracy using 20% of TP, from 0.47 to 0.43 and from 0.49 to 0.44 for 50 and 75% of TP, respectively. For TKW, it dropped from 0.75 to 0.73 and from 0.76 to 0.74 for 20 and 50% of the TP, respectively, while no difference was observed for the 75% of TP between the total number of marker and 10% of it. Statistical analysis revealed no significant differences could be observed when reducing marker number and TP size for any of the two traits.

### Genomic Prediction: Importance of Relatedness Between TP and VP (*vi*)

The four populations share common parents and have hence kinship relationships (Table 1). It was therefore evaluated if it would be possible to use one population as TP for the others (VP) which have half-sibs relationships. Using TP that were full sibs to the VP resulted in good accuracy values that ranged from 0.35 to 0.47, and from 0.92 to 0.30 for GY and TKW, respectively (Table 5). When the TP was not derived from the same cross of the BP (half sibs), the accuracies drop to values close to zero or even negative (Table 5). The only acceptable case for GY with an accuracy of 0.29 was obtained when SW was used as TP for IG, but this was not true when IG was used as TP for SW (accuracy of 0.08). The same was observed for TKW, with SW as TP ensuring an accuracy of 0.22, while YG as TP dropped to 0.09 accuracy. Interestingly, the two most genetically related populations, IG and DRO (Table 1) also resulted in very poor prediction accuracies when used as TP for each other.

**TABLE 5:** Comparison of the prediction accuracies using full sibs and half sibs as training populations for grain yield and 1,000 kernel weight.

	DRO	IG	YG	SW	DRO	IG	YG	SW
		
	Grain yield				1,000-kernels weight			
DRO	0.47	−0.08	−0.11	0.07	0.76	−0.1	0.03	−0.26
IG	−0.09	0.41	0	0.08	−0.08	0.92	−0.02	0.09
YG	−0.07	−0.02	0.35	−0.08	0.12	0	0.83	0.14
SW	0.06	0.29	−0.13	0.37	−0.26	0.22	0.11	0.3

*The columns represent the TP and the rows are the BP, the diagonal represents the full sibs relationships.*

### Genomic Prediction: Effect of QTL Analysis on Model Accuracy (*vi, viii*)

**FIGURE 5:**
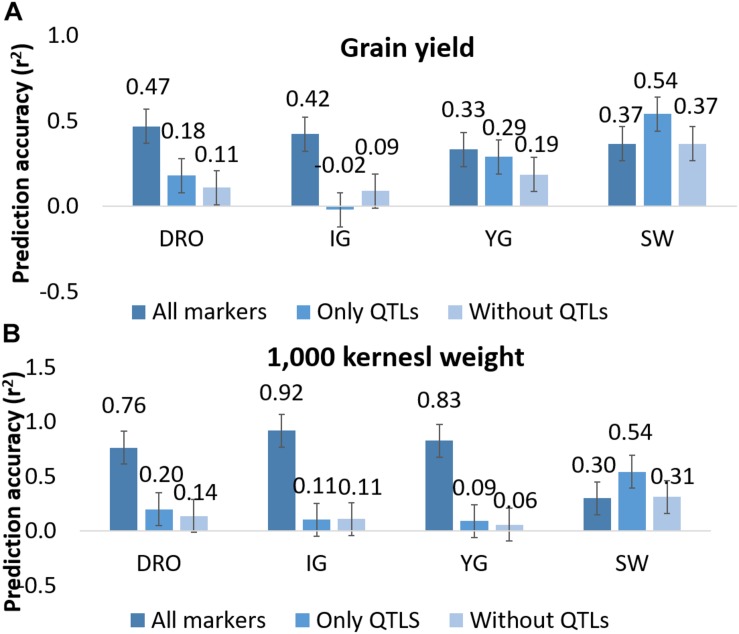
Prediction accuracy for grain yield **(A)** and 1,000 kernel weight **(B)** using all markers, only markers linked to QTLs, and all markers except those identified as linked to QTLs. Whiskers represents the experiment wise LSD.

Since QTL analysis and GS have been rarely combined, the last objective of this study was to determine if a step of QTL analysis could help improve the GS model’s accuracy. A total of 44 and 27 markers were associated via QTL analysis to GY and TKW, respectively (Figure 2). To test their value alone, these were used as the only marker to perform genomic predictions and resulted in non-significant accuracies for GY for DRO (0.18), and IG (−0.02), while significant accuracies could be identified for YG (0.29), while an increased was observed for SW (0.54). Similarly, for TKW there was a loss significance for DRO (0.20), IG (0.11) and YG (0.09), while it again increased for SW (0.54) (Figure 5). The opposite attempt was also conducted by removing from the whole set all the markers associated with QTLs. In this case the GY and TKW accuracies became non-significant for all populations, except for SW for which it matched what was obtained when using the full marker dataset (Figure 5). With the exception of SW, for which the use of only markers associated to QTLs had a positive effect on the prediction accuracies, in all other populations the use of all markers combined was significantly superior.

**FIGURE 6:**
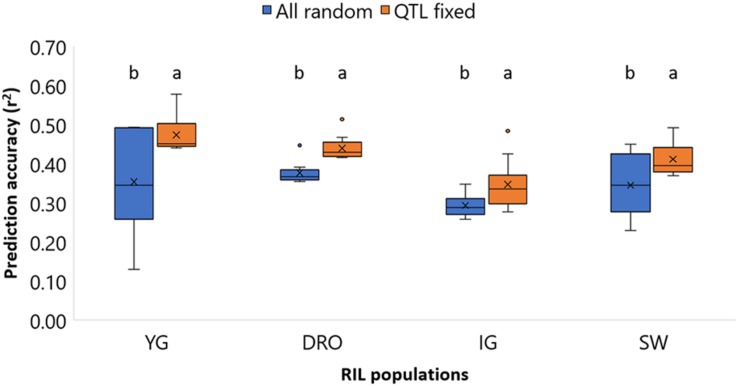
Comparison of the prediction accuracies of grain yield (GY) for the four population YG, DRO, IG, and SW, using a model with all markers considered as random effect against a models that fixed markers underlying QTLs. The horizontal line represents the average, the square indicates the 2nd and 3rd quartiles, the whiskers represent the 1st and 4th quartiles, the cross the median, and the dots are outliers. The letters indicated classes determined via LSD.

As it can be expected, the sum of the accuracies of using markers associated to large and small effects does not equal to the accuracy of these combined. It then becomes interesting to assess a model that better incorporates these two by fixing the effect of markers associated to QTLs, while including the random effect of the small impact alleles (viii). To test the suitability to do so in a context that better resembles an actual breeding pipeline, QTL discovery was re-run for each random group of entries composing the TP, and only QTL that could be identified by the specific TP where fixed in the model. Supplementary Table S4 reports how frequently the QTL associated with GY could be re-identified for each TP sub-set. The results of fixing the marker underlying the QTLs in the model is reported in Figure 6. For all four populations the accuracies increased significantly (*p* < 0.05) when the QTL-underlying markers were fixed in the model. The average accuracies shifted from 0.35 to 0.47, 0.38 to 0.44, 0.29 to 0.35, and 0.35 to 0.41, for the YG, DRO, IG, and SW populations, respectively. This represents a clear gain of 0.06–0.12 points of accuracy, superior than the 0.01–0.03 obtained by testing different statistical models (*i, ii, iii, iv*).

## Discussion

Rapid genetic gain for complex traits via traditional breeding selection is hampered by the difficulty of effectively controlling GxE in the field. Diverting the selection to the use of molecular markers promises to overcome this issue, if adequate models can be defined. Therefore, in our study we deployed four RILs populations that represented well a typical durum wheat breeding program to test the feasibility of replacing phenotypic selection with molecular selection. The four populations showed transgressive segregation when phenotyped for GY and TKW, indicating additive effect loci are present from both parents as it would be expected from a well-designed breeding cross.

### A Reliable Consensus Map

To construct a high-density consensus genetic map, a combination of four genetic backgrounds was used by anchoring common markers, followed by imputation of the missing haplotypes. The consensus map of IC, DRO, SW, YG included 14 linkage groups and spanned 2,705 cM, similar to what defined in the four way cross NCCR population map (2,664 cM) of Milner et al. (2016), and the six elite × elite populations durum wheat consensus map (2,631 cM) presented by Maccaferri et al. (2015) and in agreement with other reports ranging from 1,352 cM to 3,598 cM (Blanco et al., 1998; Nachit et al., 2001; Elouafi and Nachit, 2004; Mantovani et al., 2008; Peleg et al., 2008; Patil et al., 2013). The consensus map length was higher by 34% of the average length of the four individual maps. In agreement with previous studies (Nachit et al., 2001; Elouafi and Nachit, 2004; Peleg et al., 2008; Patil et al., 2013) and contrary to Maccaferri et al. (2015), the A and B genomes had different map lengths, with the B genome (1,572 cM) being longer than A genome (1,133.8 cM). However, similarly to Maccaferri et al. (2015), a smaller number of markers was mapped to the A genome (1,104) compared to the B genome (2,098). The marker density in the consensus map differed along the chromosomes. According to previous studies (Erayman et al., 2004; Saintenac et al., 2011; Maccaferri et al., 2015), this is probably due to the variation of recombination frequency and the potential to accumulate genetic diversity. Markers gaps of 10–33 cM were identified in all chromosomes, except chromosome 4A. Chromosome regions with reduced marker density in 1A, 2A, 3A, and 7A have also been reported in the consensus map of Maccaferri et al. (2014). Overall, the consensus map developed was well in line with previous reported examples and it was hence deemed adequate to perform the targeted study.

### Identification of Major Effect Alleles by QTL Analysis

A total of 31 QTLs were identified for DTH, DTM, PLH, TKW, SPK, and GY, with most of them showing co-localization or pleiotropic effect. Consistent QTLs for GY were detected on chromosomes 2B (Qicd.TKW.DTH.GY.001, Qicd.GY.001, Qicd.GY.002, and Qicd.TKW.GY.001), 4A (Qicd.PLH.GY.001 and Qicd.PLH.GY.002), 4B (Qicd.PLH.GY.003), 5B (Qicd.GY.003 and Qicd.PLH.GY.004), 7A (Qicd.GY.004) and 7B (Qicd.GY.005). Chromosome 2B carries 10 individual QTLs, eight of which were found associated with GY, TKW, and SPK, explaining up to 33.4% of the phenotypic variance. This is in agreement with previous reports on QTLs identified on chromosome 2B associated with GY and its components (Huang et al., 2003; McCartney et al., 2005; Quarrie et al., 2005; Suenaga et al., 2005; Huang et al., 2006; Marza et al., 2006; Maccaferri et al., 2008; Golabadi et al., 2011). Six individual QTLs for TKW were found on chromosomes 1B, 4B, 6A, 6B, and 7A. Except for Qicd.TKW.006 on 7A, which we deem to have been reported here for the first time, the five remaining QTLs have been reported in previous studies by Blanco et al. (2011) and Patil et al. (2013). As indicated by Soriano et al. (2017), QTL influencing SPK were located on chromosomes 2B, 3B, and 5B. Assanga et al. (2017) have also found in winter wheat regions in 1A and 6B that are associated with the same trait.

Major genes associated with phenology were found to have a pleiotropic influence on trait measurement and QTL detection (Acuña-Galindo et al., 2015). Flowering time is a major trait in plant breeding and it provides the basis for plant adaptation. Chromosomes 2A, 2B, 4B, 5B, 6B, and 7B harbored QTLs linked to phenology traits. On 2A and 2B, two clusters of QTLs (Qicd.DTM.PL H.TKW.DTH.001 and Qicd.TKW.DTH.GY.001) were found in approximately the same position corresponding with Ppd-A1 and Ppd-B1 genes defined by several authors (Laurie, 1997; Maccaferri et al., 2008; Wilhelm et al., 2009; Maccaferri et al., 2011; Arjona et al., 2018). In our study, GY was associated to PLH in four QTLs located on chromosomes 4A, 4B, and 5B. Previous studies have also found that PLH genes are strongly associated with QTL for GY and its components (Quarrie et al., 2005; Crossa et al., 2007; Rebetzke et al., 2008; Acuña-Galindo et al., 2015). Borner et al. (2002), Huang et al. (2003, 2006), Blanco et al. (2012), and Patil et al. (2013) found that the short arm of chromosome 2A and its homologous harbor QTL influencing TKW, that was the case for clusters Qicd.DTM.PLH.TKW.DTH.001 and Qicd.TKW.DTH.GY.001. The cluster Qicd.DTM.PLH.TKW.DTH.001 for DTM, DTH, PLH (Soriano et al., 2017) and TKW on chromosome 2A confirms its agronomically important traits contribution as reported in Maccaferri et al. (2011) and Patil et al. (2013). On the homologous region on 2B, the cluster Qicd.TKW.DTH.GY.001 influences DTH, TKW and GY. On chromosome 5B cluster Qicd.DTH.PLH.001 could be related to Vrn-B1 as reported by Hanocq et al. (2004). On the long arm of chromosomes 2B, 4B, 6B, and 7B, the identified QTLs suggest important new regions controlling earliness. Soriano et al. (2017) have also identified a novel QTL on chr. 4B and 7B. In summary, the QTL analysis of these four populations has identified and validated several previously known loci and supports their use for molecular selection.

### Selection of the Best Fitting Statistical Models for Genomic Predictions (*i, ii, iii, iv*)

The prediction analysis was conducted on the RILs population using models that account for the relationship matrix (G), environment effect (E), genotype by environment interaction (GxE), markers (M), and marker by environment interaction (MxE). The accuracy of breeding selection using only phenotypic data was computed (Figure 3) as G+E and GxE models (*i* and *ii*), to confirm that accuracies of 0.47-0.28 could be obtained via traditional breeding selection for GY. These results confirm what was reported by Crossa et al. (2014): that pedigree (population structure) accounts for a sizeable proportion of the prediction accuracy. These values were set as competitors to determine the success of replacing phenotypic selection with molecular selection. Interestingly, the GS models that incorporated marker effect (*iii, iv*) generated non-significantly different or superior accuracies than traditional breeding selection, indicating a strong role for GS in future breeding (Figure 3).

### Size and Relatedness of the Training Population (*v, vi*)

Beside academical studies, breeders often have limited resources and tend to reduce costs whenever possible. A decrease in the size of the TP that needs to be both genotyped and phenotyped, and in the number of markers to be used for genotyping can represent important savings (Heffner et al., 2011; Crossa et al., 2014; Bassi et al., 2016). This possibility was tested by varying the proportion of individuals included in TP and VP from 75% TP and 25% VP, which is a very conservative and costly approach, to 50% TP and 50% VP, and even 25% TP and 75% VP. Interestingly, non-significant differences in accuracies could be observed for any of the reductions, for both high and low heritability traits (GY and TKW).

The relatedness between the TP and VP has been identified as a key consideration for predicting complex trait with low heritability. In an ideal scenario, breeders would like to accumulate information for a TP over time, using their normal yield trials as the source for this activity. By logic, the relatedness between such a TP and a BP under selection should be that of half-sibs. To test the feasibility of this approach, the four RIL populations that share half sib relationships were used to predict each other (Table 5). This resulted in severe losses of accuracy, reaching values close to zero for both high and low heritable traits (GY and TKW). This is in agreement with Windhausen et al. (2012), who also encountered accuracies close to zero when predicting far-related populations. The relatedness of a TP to the population to be predicted is hence one of the most critical aspect of GS in durum wheat. Therefore, small TP can be effectively deployed to accurately select BP only if these have full sibs relationships with the population to be selected. This is in good agreement with Bassi et al. (2016), who described several breeding schemes to deploy GS in a manner that would allow the TP to be full-sib of the BP under selection, without excessive loss of genetic gain.

### Does Markers Number Affect the Predictions? (*v*)

The possibility of deploying GS in breeding is still heavily hindered by the cost associated with genotyping huge populations. A way to reduce the cost of genotyping would be to reduce the number of markers used for the analysis. Here we tested the effect of the markers number to reveal that there was no significant difference in the prediction accuracies between using 3,202 or 320 SNPs as far as the TP and VP are full sibs (Figure 4). Hickey et al. (2014) also reported that when using information from related maize bi-parental populations high accuracies can be achieved using a small number of markers. Similarly, Haile et al. (2018) indicated that among advanced durum wheat breeding lines, the reduction from 9,000 to 500 markers did not cause a significant reduction in accuracies. However, it has to be noted that combining a decrease of TP size to 20% of the BP, and 10% of markers number caused the accuracy for GY to drop from 0.48 to 0.41 and for TKW from 0.77 to 0.74. This is a significant reduction of 0.07 and 0.03 points. Still, in the optic of practical application, the values of accuracies remain very close to what achieved using only phenotypic models (G+E and GxE) and hence it could be advisable to deploy small TP and small markers set in breeding if this makes GS a more affordable approach.

### Is There an Advantage to Conduct QTL Analysis Before Genomic Predictions? (*vii, viii*)

QTL analysis and GS models rely on the same type of dataset. Therefore, it is of interest to define if there is additive contribution in combining both type of studies. Initially it was tested the effect of using only markers underlying QTLs to make prediction, as a way to simulate a MAS approach (Figure 5). The obtained accuracies reached between −0.02 and 0.54, depending on traits and populations. This would suggest that running prediction models using only few markers linked to known genes (44 and 27 for GY and TKW, respectively) could provide some degree of success. For confirmation, the opposite situation was also tested by removing any markers associated to QTL from the whole dataset. Once again, the accuracies dropped significantly for all traits and populations, except for SW. This result suggests that the marker number is not the only factor to ensure high accuracies, but that the ability to define the haplotype of major effect loci is also of critical importance.

The final test was designed to combine the extra information obtained via the definition of major allele effects by QTL analysis with the minor allele effects assessed via GS. Since the initial QTL discovery was conducted using the whole population, while GS models would instead use only sub-set of each population as TP and VP, QTL discovery was re-conducted for each TP subset. All initially identified QTLs were re-identified in 10–50% of the TP subsets (Supplementary Table S4) depending on the levels of allelic and phenotypic variation of each random subset. The marker underlying the re-identified QTLs were fixed for each TP subset and used to improve the prediction model. The results are extremely promising, since for all populations the combination of minor allele effects as GS random factor and major allele effects as QTL fixed factor resulted in a significant increase in prediction accuracies. Furthermore, the accuracies value were increased by 0.06–0.12 points, a major increase compared to the 0.02 points of reducing the TP size or changing statistical models. Our results are in partial agreement with Sarinelli et al. (2019) who demonstrated that major genes added as fixed effects always improved model predictive ability, with the greatest gains coming from combinations of multiple genes for days to heading and plant height in a winter wheat panel. Bian and Holland (2017) also concluded that adding SNPs associated with a given trait as fixed effects resulted in higher predictive abilities when compared to models that only treated SNPs as random effects. Bernardo (2014) pointed out that the prediction accuracy of GS models can be increased by adding major genes as fixed effects when they represent a large proportion of the total variance associated with the trait under consideration (≥10%). Considering that GY remains often the main targeted trait, and also one of the most complex to predict, overall our results support the principle of incorporating fixed effect alleles into a prediction model, especially for markers accounting for a large part of the phenotypic variation. The idea of combining MAS using marker associated to known loci as fixed effects, and all other loci as random effect, becomes interesting for practical breeding applications. Furthermore, there appears to be an additive value in conducting a discovery step via QTL analysis before running genomic predictions, since the additional information can be strategically exploited to increase accuracies.

## Conclusion

The results of this study provide a framework for better understanding and deploying molecular selection in durum wheat. The use of four populations to define a consensus linkage map allowed the precise identification of significant QTL for agronomic traits. Furthermore, these were incorporated into prediction models to reveal significant gains of accuracy for GY when integrated as fixed effects. Several critical considerations were also tested for their deployment in durum wheat breeding. The results presented here are in good agreement with previous literature and what suggested previously by us for breeding application of GS in wheat (Bassi et al., 2016). In practice, the use of half sibs or distantly related TP does not appear to be an exploitable methodology for GS in durum wheat. Instead, small size full sibs TP needs to be deployed and genotyping costs can be reduced by using just 200–300 SNPs. In addition, known loci linked to traits of interest should be also included in the marker set and used as fixed effects to increase prediction. Most importantly, all genomic prediction models were compared to the accuracy attainable by classical phenotypic selection to confirm that the same results could be achieved via molecular approaches. Altogether, our result provides strong support for the deployment of genomic prediction in durum wheat breeding.

## Data Availability Statement

The germplasm described here is available through ICARDA’s genebank and can be requested here: https://www.genesys-pgr.org/wiews/SYR002. The genotypic and phenotypic data have been provided as Supplementary Data Sheet 1.

## Author Contributions

MZ, HK, FB, ZK, and GG analyzed the data. AF-M, BB, and MN provided insightful revision and discussions. MZ, HK, FB, and MN produced the data. MZ, HK, and FB wrote the manuscript. All authors reviewed the manuscript.

## Conflict of Interest

The authors declare that the research was conducted in the absence of any commercial or financial relationships that could be construed as a potential conflict of interest.

## Supplementary Material

FIGURE S1 Cartographic location of research stations used for this study. Source: modified from Google Map.

TABLE S1 Description of the four individual maps issued from IC, DRO, SW and YG populations.

TABLE S2 Significant QTLs with LOD and phenotypic variance (PV) for the studied traits across environments of the four populations.

TABLE S3 Significant QTLs with LOD and phenotypic variance (PV) for GY, TKW, SPK and PLH of the four populations.

TABLE S4 Frequency of re-identifying QTL associated to grain yield in the ten subset of training population.

DATA SHEET S1 Complete dataset, including phenotyping information for each mapping population presented as BLUEs per location, and full genotyping file for the consensus map.
