# Supplementary figures and images for: Combining QTL Analysis and Genomic Predictions for Four Durum Wheat Populations Under Drought Conditions
Source: Front Genet. 2020 May 6;11:316. doi: 10.3389/fgene.2020.00316 (PMC7218065; doi:10.3389/fgene.2020.00316)

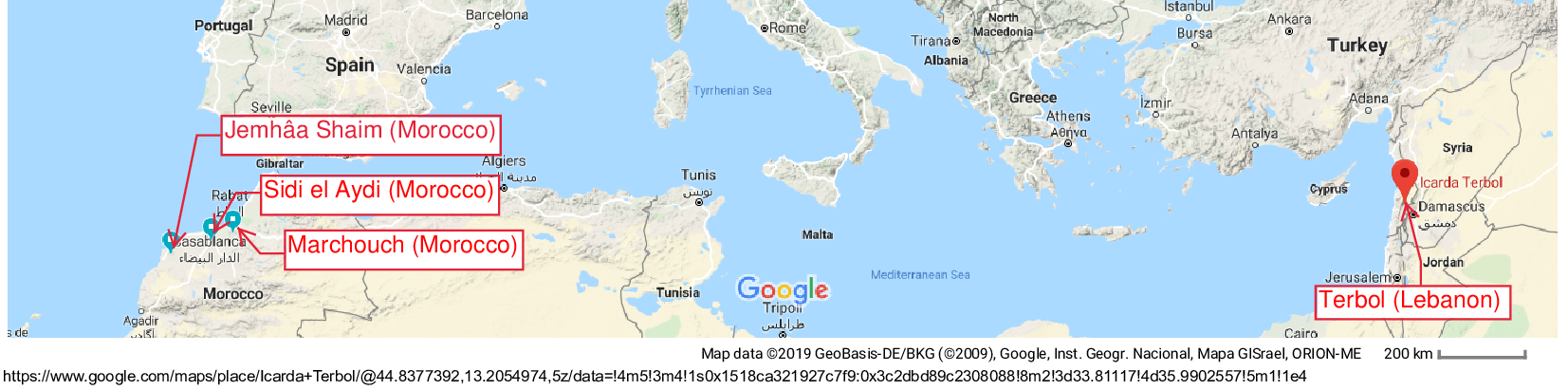

Supplement: FIGURE S1 — Cartographic location of research stations used for this study. Source: modified from Google Map. [file Image_1.TIFF]
