# Supplementary material for: Combining QTL Analysis and Genomic Predictions for Four Durum Wheat Populations Under Drought Conditions
Source: Front Genet. 2020 May 6;11:316. doi: 10.3389/fgene.2020.00316 (PMC7218065; doi:10.3389/fgene.2020.00316)
Supplement: TABLE S1 — Description of the four individual maps issued from IC, DRO, SW and YG populations. [file Data_Sheet_2.docx]

SUPPLEMENTARY MATERIAL

**Table S1**: Description of the four individual maps issued from IC, DRO, SW and YG populations.

| Chr. | IC | | |  | DRO | | |  | SW | | |  | YG | | |
| --- | --- | --- | --- | --- | --- | --- | --- | --- | --- | --- | --- | --- | --- | --- | --- |
|  | NM | LGL | MD |  | NM | LGL | MD |  | NM | LGL | MD |  | NM | LGL | MD |
| 1A | 10 | 83.3 | 9.3 |  | 91 | 60.6 | 0.7 |  | 51 | 76.4 | 1.5 |  | 11 | 145.8 | 14.6 |
| 1B | 71 | 138.2 | 2.0 |  | 171 | 132.2 | 0.8 |  | 96 | 130.3 | 1.4 |  | 64 | 200.1 | 3.2 |
|  |  |  |  |  |  |  |  |  |  |  |  |  |  |  |  |
| 2A | 14 | 20.9 | 1.6 |  | 201 | 103.1 | 0.5 |  | 28 | 88.3 | 3.3 |  | 21 | 260.0 | 13.0 |
| 2B | 152 | 278.2 | 1.8 |  | 388 | 195.0 | 0.5 |  | 186 | 165.6 | 0.9 |  | 86 | 236.8 | 2.8 |
|  |  |  |  |  |  |  |  |  |  |  |  |  |  |  |  |
| 3A | 26 | 33.7 | 1.3 |  | 59 | 210.4 | 3.6 |  | 98 | 155.3 | 1.6 |  | 19 | 35.4 | 2.0 |
| 3B | 78 | 156.2 | 2.0 |  | 199 | 118.1 | 0.6 |  | 102 | 235.4 | 2.3 |  | 45 | 169.2 | 3.8 |
|  |  |  |  |  |  |  |  |  |  |  |  |  |  |  |  |
| 4A | 54 | 113.8 | 2.1 |  | 155 | 55.7 | 0.4 |  | 94 | 86.3 | 0.9 |  | 58 | 128.3 | 2.3 |
| 4B | 30 | 81.4 | 2.8 |  | 66 | 112.3 | 1.7 |  | 89 | 112.3 | 1.3 |  | 105 | 92.1 | 0.9 |
|  |  |  |  |  |  |  |  |  |  |  |  |  |  |  |  |
| 5A | 4 | 90.1 | 30.0 |  | 98 | 204.2 | 2.1 |  | 39 | 126.6 | 3.3 |  | 4 | 51.4 | 17.1 |
| 5B | 42 | 177.3 | 4.3 |  | 258 | 178.6 | 0.7 |  | 100 | 122.6 | 1.2 |  | 6 | 84.5 | 16.9 |
|  |  |  |  |  |  |  |  |  |  |  |  |  |  |  |  |
| 6A | 64 | 182.9 | 2.9 |  | 120 | 162.6 | 1.4 |  | 110 | 116.0 | 1.1 |  | 24 | 41.3 | 1.8 |
| 6B | 41 | 87.8 | 2.2 |  | 202 | 115.3 | 0.6 |  | 49 | 192.8 | 4.0 |  | 11 | 4.5 | 0.5 |
|  |  |  |  |  |  |  |  |  |  |  |  |  |  |  |  |
| 7A | 21 | 192.2 | 9.6 |  | 69 | 133.8 | 2.0 |  | 93 | 66.0 | 0.7 |  | 50 | 190.4 | 3.9 |
| 7B | 39 | 84.2 | 2.2 |  | 214 | 140.7 | 0.7 |  | 77 | 121.5 | 1.6 |  | 17 | 44.0 | 2.8 |
|  |  |  |  |  |  |  |  |  |  |  |  |  |  |  |  |
| A genome | 193.0 | 716.9 | 8.1 |  | 793.0 | 930.5 | 1.5 |  | 513.0 | 714.8 | 1.8 |  | 187.0 | 852.6 | 7.8 |
| B genome | 453.0 | 1003.3 | 2.5 |  | 1498.0 | 992.1 | 0.8 |  | 699.0 | 1080.5 | 1.8 |  | 334.0 | 831.2 | 4.4 |
| Total | 646.0 | 1720.1 | 5.3 |  | 2291.0 | 1922.5 | 1.2 |  | 1212.0 | 1795.3 | 1.8 |  | 521.0 | 1683.8 | 6.1 |
|  |  |  |  |  |  |  |  |  |  |  |  |  |  |  |  |

NM: number of markers mapped on each chromosome.

MD: average marker density in cM per marker.

LGL: linkage group length in cM.

**Table S2**: Significant QTLs with LOD and phenotypic variance (PV) for the studied traits across environments of the four populations

| Chr | QTL ID | Trait | Population | Marker | Interval size (cM) | LOD | PV (%) |
| --- | --- | --- | --- | --- | --- | --- | --- |
| 2A | Qicd.DTM.PLH.TKW.DTH.001 | DTM | SW | Icr_4933 | 2.2 | 20.8 | 47.6 |
|  |  | PLH | SW | Icr_4933 | 2.2 | 20.8 | 47.6 |
|  |  | TKW | SW | Icr_4933 | 2.2 | 20.8 | 47.6 |
|  |  | DTH | SW | Icr_4647 | 0.4 | 43.5 | 81.3 |
| 2B | Qicd.SPK.DTM.001 | SPK | YG | c2B.loc28 | 3.9 | 3.7 | 7.9 |
|  |  | DTM | IC | Icr_13299 | 3.9 | 4.3 | 9.2 |
|  | Qicd.TKW.DTH.GY.001 | TKW | YG | Icr_12395 | 1.5 | 9.6 | 20.5 |
|  |  | DTH | YG | c2B.loc48 | 1.2 | 10.9 | 33.4 |
|  |  | GY | YG | c2B.loc48 | 8.6 | 10.9 | 33.4 |
|  | Qicd.DTM.001 | DTM | YG | c2B.loc152 | 4.6 | 7.7 | 16.9 |
| 4B | Qicd.TKW.DTM.001 | TKW | SW | c4B.loc160 | 1.7 | 4.8 | 10.0 |
|  |  | DTM | IC | Icr_17201 | 0.8 | 4.8 | 8.2 |
| 5B | Qicd.DTH.PLH.001 | DTH | IC | c5B.loc18 | 1.9 | 5.4 | 12.5 |
|  |  | PLH | IC | c5B.loc21 | 2.3 | 4.5 | 11.5 |
| 6B | Qicd.DTM.002 | DTM | SW | Icr_15992 | 0.6 | 6.8 | 13.8 |
| 7B | Qicd.DTH.001 | DTH | IC | c7B.loc138 | 3.1 | 3.8 | 8.7 |
|  |  |  |  |  |  |  |  |

**Table S3**. Significant QTLs with LOD and phenotypic variance (PV) for GY, TKW, SPK and PLH of the four populations.

| Chr | QTL ID | Trait | Population | Marker | Position (cM) | LOD | PV (%) |
| --- | --- | --- | --- | --- | --- | --- | --- |
| 1A | Qicd.SPK.001 | SPK | IC | Icr_6218 | 111.3 | 5.3 | 8.0 |
| 1B | Qicd.TKW.001 | TKW | IC, YG | Icr_6388 | 34.4 | 4.0 | 7.7 |
|  | Qicd.TKW.002 | TKW | IC | c1B.loc125 | 124.4 | 6.1 | 15.9 |
| 2B | Qicd.GY.001 | GY | YG | Icr_7246 | 69.3 | 5.0 | 13.4 |
|  | Qicd.GY.002 | GY | IC | Icr_18720 | 107.0 | 4.4 | 6.2 |
|  | Qicd.TKW.GY.001 | TKW | SW | c2B.loc125 | 123.7 | 4.7 | 8.6 |
|  |  | GY | YG | Icr_687 | 135.8 | 4.3 | 4.8 |
| 3B | Qicd.SPK.002 | SPK | SW | Icr_5915 | 66.5 | 5.3 | 13.2 |
| 4A | Qicd.PLH.GY.001 | PLH | DRO | Icr_21319 | 20.6 | 4.0 | 4.2 |
|  |  | GY | IC | c4A.loc33 | 32.9 | 3.8 | 5.1 |
|  | Qicd.PLH.GY.002 | GY | SW | Icr_17289 | 43.8 | 6.2 | 19.3 |
|  |  | PLH | SW | Icr_1963 | 50.9 | 8.1 | 14.1 |
| 4B | Qicd.PLH.001 | PLH | DRO | c4B.loc19 | 13.4 | 6.3 | 10.0 |
|  | Qicd.PLH.GY.003 | GY | YG | Icr_987 | 32.9 | 7.5 | 9.4 |
|  |  | PLH | YG | Icr_7052 | 40.3 | 11.7 | 32.7 |
|  | Qicd.TKW.003 | TKW | DRO, YG | Icr_16461 | 49.1 | 4.5 | 9.3 |
| 5B | Qicd.GY.003 | GY | YG | Icr_15349 | 101.9 | 4.9 | 7.3 |
|  | Qicd.PLH.GY.004 | PLH | DRO | c5B.loc116 | 112.9 | 4.0 | 3.9 |
|  |  | GY | DRO | c5B.loc121 | 116.9 | 4.1 | 6.0 |
|  | Qicd.PLH.002 | PLH | YG | Icr_20534 | 154.1 | 6.6 | 8.4 |
|  | Qicd.SPK.003 | SPK | DRO | Icr_2977 | 195.8 | 6.1 | 6.6 |
| 6A | Qicd.TKW.004 | TKW | YG | Icr_15049 | 97.2 | 4.1 | 9.5 |
| 6B | Qicd.TKW.005 | TKW | YG | c6B.loc6 | 5.1 | 6.1 | 4.7 |
|  | Qicd.PLH.003 | PLH | YG | Icr_9314 | 79.0 | 4.5 | 15.8 |
|  | Qicd.SPK.004 | SPK | SW | Icr_17674 | 173.1 | 6.1 | 10.9 |
| 7A | Qicd.TKW.006 | TKW | DRO | Icr_5226 | 9.2 | 5.3 | 10.4 |
|  | Qicd.GY.004 | GY | DRO | Icr_16322. | 138.7 | 4.4 | 4.3 |
| 7B | Qicd.GY.005 | GY | SW | Icr_14524 | 110.6 | 3.9 | 11.7 |
|  |  |  |  |  |  |  |  |

QTL ID: Qicd(icarda).trait.number, PV: Phenotypic variance (%), significant QTL p < 0.05

**Table S4**: Frequency of re-identifying QTL associated to grain yield in the ten subset of training population.

|  | | | Frequency of QTL detection in the 10 TP subset | | | |
| --- | --- | --- | --- | --- | --- | --- |
| Chr | QTL ID | Position (cM) | DRO | IG | YG | SW |
| 2B | Qicd.GY.001 | 69.3 | 50% | - | 30% | 20% |
| 2B | Qicd.GY.002 | 107.0 | 30% | 40% | 20% | - |
| 2B | Qicd.TKW.GY.001 | 135.8 | - | - | 40% | - |
| 4A | Qicd.PLH.GY.001 | 20.6 | - | 20% | - | 20% |
| 4A | Qicd.PLH.GY.002 | 43.8 | - | - | - | 30% |
| 4B | Qicd.PLH.GY.003 | 32.9 | 10% | - | 20% | - |
| 5B | Qicd.GY.003 | 101.9 | 30% | 10% | - | 20% |
| 7A | Qicd.GY.004 | 138.7 | 20% | 20% | 10% | 20% |
| 7B | Qicd.GY.005 | 110.6 | - | 20% | - | 40% |
